# Breast cancer incidence highest in the range of one species of house mouse, *Mus domesticus*

**DOI:** 10.1054/bjoc.1999.0941

**Published:** 2000-01-18

**Authors:** T H M Stewart, R D Sage, A F R Stewart, D W Cameron

**Affiliations:** University of Ottawa at Ottawa Hospital, General Site, Ottawa, Ontario, K1H 8M5, Canada; Department of Biological Sciences, University of California, Santa Barbara, CA, 93106, USA; Department of Medicine and Graduate Program in Biochemistry and Molecular Genetics, University of Pittsburgh, PA, 15213, USA

**Keywords:** human breast cancer, mouse mammary tumour virus, zoonosis, geographic variation, incidence, *Mus domesticus*, MMTV

## Abstract

Incidence of human breast cancer (HBC) varies geographically, but to date no environmental factor has explained this variation. Previously, we reported a 44% reduction in the incidence of breast cancer in women fully immunosuppressed following organ transplantation (Stewart et al (1995) *Lancet***346**: 796–798). In mice infected with the mouse mammary tumour virus (MMTV), immunosuppression also reduces the incidence of mammary tumours. DNA with 95% identity to MMTV is detected in 40% of human breast tumours (Wang et al (1995) *Cancer Res***55**: 5173–5179). These findings led us to ask whether the incidence of HBC could be correlated with the natural ranges of different species of wild mice. We found that the highest incidence of HBC worldwide occurs in lands where *Mus domesticus* is thse resident native or introduced species of house mouse. Given the similar responses of humans and mice to immunosuppression, the near identity between human and mouse MTV DNA sequences, and the close association between HBC incidence and mouse ranges, we propose that humans acquire MMTV from mice. This zoonotic theory for a mouse-viral cause of HBC allows testable predictions and has potential importance in prevention. © 2000 Cancer Research Campaign


*British Journal of Cancer* (2000) **82**(2), 446–451
© 2000 Cancer Research Campaign
Article no. bjoc.1999.0941

### Breast cancer incidence highest in the range of one species of house mouse, *Mus domesticus*

**THM Stewart[1], RD Sage[2], AFR Stewart[3] and DW Cameron[1]**

[1]University of Ottawa at Ottawa Hospital, General Site, Ottawa, Ontario, K1H 8M5, Canada; [2]Department of Biological Sciences, University of California, Santa Barbara, CA 93106, USA; [3]Department of Medicine and Graduate Program in Biochemistry and Molecular Genetics, University of Pittsburgh, PA 15213, USA

**Summary** Incidence of human breast cancer (HBC) varies geographically, but to date no environmental factor has explained this variation. Previously, we reported a 44% reduction in the incidence of breast cancer in women fully immunosuppressed following organ transplantation (Stewart et al (1995) *Lancet* **346**: 796–798). In mice infected with the mouse mammary tumour virus (MMTV), immunosuppression also reduces the incidence of mammary tumours. DNA with 95% identity to MMTV is detected in 40% of human breast tumours (Wang et al (1995) *Cancer Res* **55**: 5173–5179). These findings led us to ask whether the incidence of HBC could be correlated with the natural ranges of different species of wild mice. We found that the highest incidence of HBC worldwide occurs in lands where *Mus domesticus* is the resident native or introduced species of house mouse. Given the similar responses of humans and mice to immunosuppression, the near identity between human and mouse MTV DNA sequences, and the close association between HBC incidence and mouse ranges, we propose that humans acquire MMTV from mice. This zoonotic theory for a mouse-viral cause of HBC allows testable predictions and has potential importance in prevention. © 2000 Cancer Research Campaign

**Keywords:** human breast cancer; mouse mammary tumour virus; zoonosis; geographic variation; incidence; *Mus domesticus*; MMTV

Human breast cancer (HBC) incidence varies worldwide (Parkin et al, 1997). People moving from areas of lower to higher HBC incidence show a gradually acquired increase in risk. For example, the Japanese moving to the USA (Ziegler et al, 1993), Soviet Jews to Israel (Iscovich and Howe, 1998) and south Asians to the UK (Winter et al, 1999) all experience increased incidences of breast cancer over a period of decades. Changes in the environment are universally agreed to account for the increased risk for breast cancer (Hunter et al, 1997). Genetic factors (inherited mutations in BRCA1 and BRCA2) account for about 5% of HBC (Szabo and King, 1997). Diets high in fat and xeno-oestrogens have been proposed to increase HBC incidence, whereas phyto-oestrogens may have a protective effect, although none of these hypotheses have shown a correlation to the migration effect (Hunter et al, 1996, 1997; Holmes et al, 1999). Moreover, the circumpolar Inuit have a low breast cancer incidence, half that in Canada (Miller and Gaudette, 1996), but have a high saturated fat diet that is low in phyto-oestrogens and is contaminated with high levels of xeno-oestrogens (Ayotte et al, 1997). Some other, unrecognized, environmental factors with oncogenic potential must explain the geographic differences in HBC incidence.

Mouse mammary tumour virus (MMTV) is an oncogenic B-type retrovirus that causes breast cancer in mice. MMTV env gene-like sequences are found in 38–40% of HBC and are 95–98% identical to MMTV over 660 bp of sequence, but not to other known human endogenous retroviruses (Wang et al, 1995). These authors have recently extended these findings by isolating 9.9 kb fragments from two human breast carcinomas containing *env, gag, pol* and LTR genes 94% homologous to MMTV, but only 90% homologous to each other (Liu et al, 1999). The presence of MMTV in HBC has been confirmed in two independent studies (Imai, 1996; Lushnikova et al, 1998). Similarity of the complete genomes among different MMTV strains in mice is also about 95% (Nishio et al, 1994), arguing that human MTVs are acquired from mice. These DNA findings lend credence to earlier reports of MMTV-like particles (Dmochowski et al, 1969) and antibody responses to MMTV proteins (Day et al, 1981) in breast tumours.

Strong support for humans becoming infected with MMTV comes from a study of laboratory personnel working with MMTV-infected mice. They were found to develop a specific serologic response to MMTV when compared to age- and gender-matched controls, most strongly to gp55, a surface glycoprotein of MMTV (Dion et al, 1986). This retrospective study was prompted by reports of immune reactivity to MMTV in exposed laboratory personnel (Holder et al, 1976; Wiseman et al, 1980; Lopez et al, 1981). Most notably was the case of a woman working with MMTV-infected mice. Over a 28-month period she was seronegative by enzyme-linked immunosorbent assay (ELISA) and indirect immunofluorescence, but at the 32nd month she seroconverted. Nine months later a small mass in her right breast was discovered, diagnosed 3 months later as an infiltrating ductal carcinoma (Poon et al, 1983). From these reports and their own study, Dion et al (1986) recommended more stringent guidelines for laboratory containment of MMTV, to prevent zoonotic transmission of the virus. The question remains how the general population acquires the MMTV detected in breast cancer specimens.

House mice, of the genus *Mus*, range naturally across Asia, Europe and North Africa, but do not inhabit the circumpolar region above the treeline (Forsyth, 1985). The evolutionary relationships and biogeography of these mice are now well understood

*Received 3 March 1999*
*Revised 13 July 1999*
*Accepted 3 August 1999*

*Correspondence to:* THM Stewart, 1 Mount Pleasant Ave, Ottawa, Ontario, Canada K1S 0L6. E-mail: tstewottcan@msn.com

(Boursot et al, 1993; Sage et al, 1993). The taxonomy for naming the evolutionary lineages of mice is contentious, some calling them three subspecies of *M. musculus* (Boursot et al, 1993), while others treat them as three separate species, namely *M. domesticus*, *M. musculus* and *M. castaneus* (Sage et al, 1993). We use the full-species taxonomy because it emphasizes the distinctiveness of these different mice. The native ranges of the three species are non-overlapping, and where they meet there are comparatively narrow zones of hybridization (Sage et al, 1993). Broadly speaking, *M. musculus* ranges from central Europe east to China and Japan, *M. castaneus* occurs from southern China to central Iran, and *M. domesticus* lives from western Iran to western Europe. *M. domesticus* mice also expanded their range to North and South America, Australia, New Zealand and Hawaii via ships sailing from western European ports. Inbred laboratory mice have mostly *M. domesticus* genes (Callahan, 1996), although some inter-breeding with *M. musculus* imported from China and Japan has occurred (Blank et al, 1986).

MMTV has been studied mostly in inbred strains of mice (Traina-Dorge and Cohen, 1983). What little is known about MMTV in wild mice suggests that it is common and exists in even greater diversity than in inbred mice (Callahan et al, 1986). The virus occurs either as independent exogenous infectious particles that cause mammary tumours or as a part of the rodent's germline genome (endogenous provirus). Exogenous virus was detected in about 50% of *M. domesticus* mice from Southern California (Rongey et al, 1973) and in 43% of *M. musculus* from Moscow (Pogossiantz, 1956). Data on the presence of exogenous virus in *M. castaneus* mice are lacking.

Mouse species differ in the numbers of endogenous MMTV proviral loci, with *M. musculus* and *M. castaneus* having the fewest and *M. domesticus* having the most ($P < 0.02$, Table 1). Laboratory colonies of feral mice have been established from different geographic locations. Descendants of these wild *M. musculus* mice collected at six Asian (Callahan et al, 1982; Imai et al, 1994) and one Czech locality had no (0) complete locus (Callahan et al, 1982; Jouvin-Marche et al, 1992). At two Asian localities (Imai et al, 1994) and two Czech localities mice had 1–2 loci, and at one Austrian locality mice had 2 loci (Jouvin-Marche et al, 1992). *M. castaneus* mice from three Asian localities had 0 locus (Taiwan), 1–2 loci (Indonesia) and 3 loci (Malaysia) respectively (Imai et al, 1994). Most wild *M. domesticus* mice assayed have been from North America, where this species was introduced from Europe. Colonies were established from feral mice from six Delaware–Maryland localities (Callahan et al, 1986), four California localities (Cohen and Varmus, 1979), and two localities in Morocco (Callahan et al, 1982). One North American and one Moroccan colony had no complete endogenous locus, 1–2 loci were found in one Moroccan and three North American colonies, and seven North American colonies had 3–5 loci. The number of endogenous loci ranges from 3 to 8 in the classical inbred strains (Kozak et al, 1987). Wild *M. domesticus* mice from Europe have apparently never been genotyped. However, the high numbers of loci in their North American descendants and in the classical lab strains suggests that European *M. domesticus* will also have higher average numbers of proviral loci than *M. musculus* mice. Since the exogenous infectious particles appear to evolve repeatedly from different endogenous viruses (Doolittle et al, 1989; Brandt-Carlson et al, 1993), and because exogenous virus can recombine with endogenous virus to generate a novel variant with broadened host range (Golovkina et al, 1994), we expect that mouse populations with more endogenous loci will have more kinds of infectious MMTV.

**Table 1** Number of feral mouse colonies for *M. musculus*, *M. castaneus* and *M. domesticus* from different localities sorted by number of endogenous MMTV copies and compared to inbred *M. domesticus* strains

| | ***M. musculus*** | ***M. castaneus*** | ***M. domesticus*** | ***M. domesticus* (inbred)** |
|---|---|---|---|---|
| ***n*** | **12** | **3** | **13** | **13** |
| MMTV copy no. | | | | |
| **0** | 7 | 1 | 2 | 0 |
| **1–2** | 5 | 1 | 4 | 0 |
| **3–8** | 0 | 1 | 7 | 13 |

Each feral mouse colony was established from at least one pair of breeding founders caught at a given locality (Cohen and Varmus, 1979; Callahan et al, 1982, 1986; Jouvin-Marche et al, 1992; Imai et al, 1994). Data for inbred strains are from Kozak et al (1987). The modal number of endogenous MMTV loci in feral *M. musculus* is 0, for *M. castaneus* is 1–2, and for *M. domesticus* is 3–8. Feral *M. domesticus* mice are significantly more likely to have 3–8 endogenous loci than the other feral mice ($P = 0.011$ by Fisher's exact test).

Since mouse species differ in their numbers of proviral loci and perhaps in the frequency of exogenous infections, we asked whether HBC incidence differs in the lands of the three species of mice.

### MATERIALS AND METHODS

The distribution in the number of endogenous MMTV loci in feral mouse colonies was compared for all known published reports and ranked in two categories: 0 complete locus and 1–2 loci in one category, and 3–8 loci in the other. The proportion of observations in the different categories was compared to the distribution expected from random occurrence using Fisher's exact test and was considered significant at the $P < 0.05$ level.

HBC incidence per 100 000 (world age-standardized incidence rate by site), taken from Parkin et al (1997) was compared between different countries grouped according to the natural range of their resident species of house mouse (Boursot et al, 1993; Sage et al, 1993). Two geographic regions were considered: western and eastern Europe, where a clear dichotomy in the native ranges of the different species of house mice exists, and the rest of the world, where *M. domesticus* has been introduced in many countries by colonization from European sailing ships. Means of HBC incidence were compared by independent samples T test using the statistical program SPSS and considered significant at the $P < 0.05$ level.

### RESULTS

In Europe there is an abrupt change in the distribution of *M. domesticus* and *M. musculus*, with a well characterized narrow hybrid zone that runs through central Europe (Sage et al, 1993). Figure 1A shows the western European distribution of *M. domesticus*, the eastern European distribution of *M. musculus* and the narrow hybrid zone (Sage et al, 1993). Figures 1B and 1C show the HBC incidence (Parkin et al, 1997) in lands of *M. domesticus* and *M. musculus*. HBC incidence is 54% higher in western

*© 2000 Cancer Research Campaign*

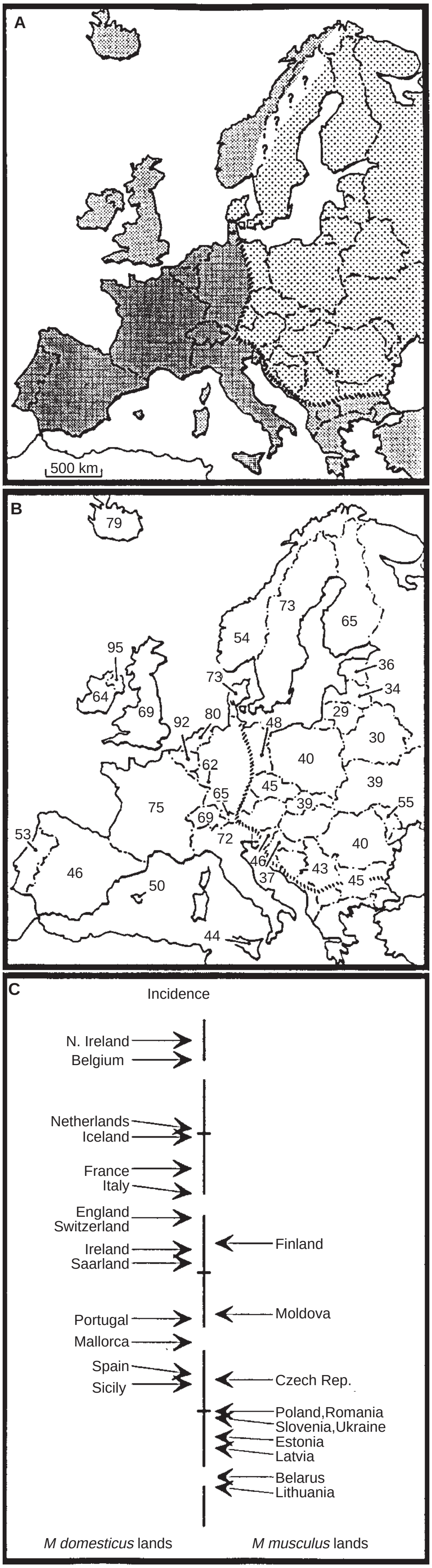

**Figure 1(A)** Western European distribution of *M. domesticus* (heavy stippling), the eastern European distribution of *M. musculus* (light stippling) and the narrow hybrid zone (hatched) where both species are found (Sage et al, 1993). The boundary of *M. domesticus* and *M. musculus* distribution is poorly known in central Scandinavia (?). (**B, C**) HBC incidence per 100 000 annotated for each country. Incidence for N. Ireland, Belgium, Portugal, Moldova, Romania, Ukraine and Lithuania are from Doll (1969). All other values are from Parkin et al (1997)

European lands of *M. domesticus* than eastern European lands of *M. musculus*. This difference is statistically highly significant ($P < 0.001$, see Table 2). HBC incidence data published in 1969 for these same countries (Doll, 1969) show that the same relative difference has been maintained over 30 years (data not shown).

Cancer incidence in Finland is higher than expected for an *M. musculus* land (Table 2). *M. musculus* mice in Finland were shown to have nuclear DNA of *M. musculus* but mitochondrial DNA of *M. domesticus* (Prager et al, 1993), suggesting that MMTV of *M. domesticus* mice might also be in Finland, explaining the high incidence in Finland.

In Table 3, HBC incidence (Parkin et al, 1997) in other lands having *M. domesticus* is compared to regions where *M. domesticus* is not found. Here too the incidence rates stay high ($P < 0.001$), even though the environments of these foreign lands vary enormously in temperature, rainfall, and native biota. The potential oncogenic factor for high HBC incidence common to most of the world is the presence of *M. domesticus* and its MMTV. We would emphasize that these strong correlations do not demonstrate cause, but demand an explanation.

### DISCUSSION

A frequently cited explanation for a high incidence of HBC is standard of living. However, the relatively high standard of living in Taiwan, Japan, Hong Kong and Singapore does not equate with a high incidence of HBC, quite the contrary. The age standardized rate (ASR) for Taiwan is 17 (Chie et al, 1995), Japan, 26; Hong Kong, 34; and Singapore, 39 (Parkin et al, 1997). In contrast, we show that a high incidence of HBC is found in the range of one mouse species.

Many parallels in mouse and HBC oncogenesis and pathology exist. First, in the mouse, MMTV itself does not carry a transforming oncogene but acts as an insertional mutagen with several proviral insertion loci (Nusse, 1991). *Wnt1* and *Wnt3* are two genes that cause mammary tumours in mice, when activated by MMTV. In women, the related genes *Wnt2*, *Wnt4* and *Wnt7b* are associated with abnormal proliferation in human breast tissue (Huguet et al, 1994). The human homologue of mouse *Wnt10b*, shown to cooperate with FGF3 in the development of MMTV-induced mouse mammary carcinomas, is upregulated in human breast carcinomas (Bui et al, 1997). These observations are compatible with the effect of an insertional mutagen in women. Second, breast cancer oncogenesis in the mouse is stimulated by T-cell immune reaction to the superantigen of MMTV (Wei et al, 1993). A direct effect of MMTV superantigen is a profound and unambiguous specific T-cell deletion, based predominantly on V$\beta$ expression. Human T-cells also respond to MMTV-encoded superantigen with V$\beta$ restriction (Labrecque et al, 1993). Third, immunosuppression decreases the incidence of murine breast cancers (Stewart and Heppner, 1997). Similarly, fully immunosuppressed women (those receiving cyclosporin, azathioprine and

 *© 2000 Cancer Research Campaign*

**Table 2** HBC incidence (Parkin et al, 1997) per 100 000 (world age-standardized incidence rate by site) in western European lands of *M. domesticus*, lands intersected by the hybrid zone, and eastern European lands of *M. musculus*

| **Lands of *M. domesticus*** | | **Lands of the hybrid zone** | | **Lands of *M. musculus*** | |
|---|---|---|---|---|---|
| Spain (9) | 46[a] | Croatia | 37 | Belarus | 30 |
| W. Germany (Saarland) | 62 | Yugoslavia | 43 | Latvia | 34 |
| Ireland | 64 | Slovenia | 46 | Estonia | 36 |
| England & Wales | 69 | E. Germany | 48 | Slovak Republic | 39 |
| Switzerland (8) | 69[a] | Norway | 54 | Poland (3) | 40[a] |
| Italy | 72 | Austria (Tyrol) | 65 | Czech Republic | 45 |
| France (8) | 75[a] | Sweden | 73 | Finland | 65 |
| Iceland | 79 | Denmark | 73 | | |
| Netherlands | 80 | | | | |
| | **64.5 ± 13.61** | | **53.6 ± 13.52** | | **41.8 ± 10.06** |
| Mean ± s.d. | | | | | |

[a]Values are weight means of incidence at multiple localities within a given country (number of localities indicated in parentheses). Difference between breast cancer incidence in lands of *M. domesticus* and *M. musculus*, $P < 0.001$, by independent-samples *t*-test.

**Table 3** HBC incidence (Parkin et al, 1997) per 100 000 (world age-standardized incidence rate by site) in lands where *M. domesticus* is the resident native or introduced species of house mouse compared to lands inhabited by *M. musculus*, *M. castaneus* and other mice

| **Lands of *M. domesticus*** | | **Lands inhabited by other mice** | |
|---|---|---|---|
| Algeria | 10 | South Korea | 7 |
| Ecuador | 27 | Thailand (2) | 12[a] |
| Costa Rica | 29 | Taiwan | 17[b] |
| Peru (2) | 31[a] | Vietnam | 18 |
| Columbia | 39 | India (5) | 21[a] |
| Brazil (3) | 44[a] | China (2) | 26[a] |
| Puerto Rico | 46 | Japan (6) | 26[a] |
| Argentina | 60 | Circumpolar Inuit | 34[c] |
| Australia | 67 | | |
| Canada | 77 | | |
| New Zealand | 77 | | |
| Israel | 77 | | |
| USA (11) | 79[a] | | |
| Uruguay (Montevideo) | 93 | | |
| Hawaii | 97 | | |
| | **66.0 ± 27.14** | | **21.7 ± 8.12** |
| Mean ± s.d. | | | |

[a]Values are weight means of incidence at multiple localities within a given country (number of localities indicated in parentheses). Difference between breast cancer incidence in lands of *M. domesticus* and other mice, $P < 0.001$, by independent-samples *t*-test. [b]Data are from Chie et al (1995). [c]Data are from Miller and Gaudette (1996). There are no mice of the genus *Mus* in the circumpolar Inuit environment (Forsyth, 1985).

steroids) show a 44% reduction in the incidence of breast cancer, but a significant increase in many other cancers (Stewart et al, 1995), supporting a parallel immune promotion of breast cancer oncogenesis in humans and mice. These observations support our hypothesis, given the finding of MMTV DNA in 38–40% of HBC (Wang et al, 1995).

The possibility of a rodent-borne virus causing this major human disease and accounting for almost half of its incidence should not be surprising nor unexpected. Almost 20 years ago, Day et al (1981) drew this same conclusion from the geographic variation in immunological responses to antigens of MMTV in the sera of patients with breast cancer. Precedence for the hypothesis that retroviruses cause human cancers exists. For example, human T-cell leukaemia virus (HTLV-1) is associated with adult T-cell leukaemia (Tajima and Cartier, 1995). There is evidence that HTLV-1 was acquired by zoonotic infection from rats and mice (Fukui et al, 1983). *M. domesticus* has lived with humans, as an intimate commensal, since the beginnings of agricultural societies in the Near East (Auffray et al, 1988). The existence of regulatory food standards allowing up to two pellets of rodent excreta per pint of wheat (US pt = 551 $cm^3$) confirm the presence of mice in the modern human food chain (Gecan et al, 1980).

A number of ‘new’ human diseases associated with rodents have recently been described (Mills and Childs, 1998). Many major human diseases have shifted to us from our domesticated animals. Antibodies to intracytoplasmic A-type particles of MMTV have been found in domesticated mammals (Zotter, 1983), suggesting cross-species infectivity of MMTV. MMTV has been shown to replicate in cultured cells of rat, cat, dog, mink and human breast cancer (Lasfargues et al, 1976*a*, 1976*b*, 1979; Ringold et al, 1977; Howard and Schlom, 1980). Breast-fed infants of mothers who subsequently develop breast cancer are not at higher risk of developing the disease (Titus-Ernstoff et al, 1998), suggesting that an oncogenic human virus is not vertically

© 2000 Cancer Research Campaign 

transmitted from mother to infant. However, these results do not exclude a zoonotic hypothesis of MMTV acquired with every new generation. Molecular and geographic evidence is becoming an acceptable alternative to Koch's postulates for identifying pathogens of new diseases (Mills and Childs, 1998). The co-occurrence of the highest incidence of HBC in the presence of *M. domesticus* leads to testable predictions.

### Prediction 1

The proportion of tumours testing positive for MMTV DNA should be lowest in *M. musculus* and *M. castaneus* lands. Tentative support of this prediction already exists: DNA studies of histological sections (Haga et al, 1994) and immunological studies (Day et al, 1981) found signs of MMTV in only 5% of patients in Asian countries. The report (Wang et al, 1995) of 38–40% of tumours positive for MMTV were from North American patients, living in the land of *M. domesticus*. Moreover, the immunosuppressed transplant patients showing a 44% reduction in breast cancer incidence are from western Europe and North America (Stewart et al, 1995). A similar high incidence of MMTV DNA is expected in tumours from patients living in western Europe, South America, Australia, New Zealand and Hawaii.

### Prediction 2

People who live and work where *M. domesticus* is especially common should have a higher MMTV seroprevalence than those not so directly exposed to mice. A cross-sectional and longitudinal sero-epidemiological study of women attending mammography screening clinics should be conducted. We predict that MMTV seronegative women will have a lower prevalence and incidence of HBC compared with MMTV seropositive women.

### Prediction 3

Tissues from human tumours testing positive for MMTV should produce mammary tumours when injected into uninfected mice, while MMTV-negative human tumours should not. Significantly, the feasibility of this approach has already been demonstrated by the experiments of Medvedev in the early 1950s, who was able to cause uninfected mice to develop mammary tumours when injected with extracts from human breast tumours, as cited in (Pogossiantz, 1956).

If the higher incidence of HBC in lands of *M. domesticus* reflects oncogenic MMTV zoonosis, then this raises the real possibility of reducing breast cancer incidence by the development of an MMTV vaccine.

### ACKNOWLEDGEMENTS

We thank A Badley, R Callahan, JW Carnwath, D Hölzel, J Mills, N Sarkar and C Vutuc for helpful discussion and ideas, and the following for their invaluable help: N Birkett, D Cameron, DA Enria, F Furmankiewicz, G Laidlaw, C Pasqualini, J Renaud-Skuce, V Schusterman, N Stewart, B Theriault, N Webb and L Woodcock. DW Cameron was supported by a Career Scientist Award of the Ontario Ministry of Health. Travel support was provided by International BioImmune Systems, Inc., Great Neck, New York.

### REFERENCES

Auffray J-C, Tchernov E and Nevo E (1988) Origine du commensalisme de la souris domestique (*Mus musculus domesticus*) vis-à-vis de l'homme. *Comptes Rendus Acad Sci* **307:** 517–522

Ayotte P, Dewailly E, Ryan JJ, Bruneau S and Lebel G (1997) PCBs and dioxin-like compounds in plasma of adult Inuit living in Nunavik (Arctic Quebec). *Chemosphere* **34**: 1459–1468

Blank RD, Campbell GR and D'Eustachio P (1986) Possible derivation of the laboratory mouse genome from multiple wild *Mus* species. *Genetics* **114**: 1257–1269

Boursot P, Auffray JC, Britton-Davidian J and Bonhomme F (1993) The evolution of house mice. *Annu Rev Ecol Syst* **24**: 119–152

Brandt-Carlson C, Butel JS and Wheeler D (1993) Phylogenetic and structural analyses of MMTV LTR ORF sequences of exogenous and endogenous origins. *Virology* **193**: 171–185

Bui TD, Rankin J, Smith K, Huguet EL, Ruben S, Strachan T, Harris AL and Lindsay S (1997) A novel human Wnt gene, WNT10B, maps to 12q13 and is expressed in human breast carcinomas. *Oncogene* **14**: 1249–1253

Callahan R (1996) MMTV-induced mutations in mouse mammary tumors: their potential relevance to human breast cancer. *Breast Cancer Res Treat* **39**: 33–44

Callahan R, Drohan W, Gallahan D, D'Hoostelaere L and Potter M (1982) Novel class of mouse mammary tumor virus-related DNA sequences found in all species of *Mus*, including mice lacking the virus proviral genome. *Proc Natl Acad Sci USA* **79**: 4113–4117

Callahan R, Gallahan D, D'Hoostelaere LA and Potter M (1986) Endogenous MMTV proviral genomes in feral *Mus musculus domesticus. Curr Top Microbiol Immunol* **127**: 362–370

Chie WC, Chen CF, Chen CJ, Chang CL, Liaw YP and Lin RS (1995) Geographic variation of breast cancer in Taiwan: international and migrant comparison. *Anticancer Res* **15**: 2745–2749

Cohen JC and Varmus HE (1979) Endogenous mammary tumour virus DNA varies among wild mice and segregates during inbreeding. *Nature* **278**: 418–423

Day NK, Witkin SS, Sarkar NH, Kinne D, Jussawalla DJ, Levin A, Hsia CC, Geller N and Good RA (1981) Antibodies reactive with murine mammary tumor virus in sera of patients with breast cancer: geographic and family studies. *Proc Natl Acad Sci USA* **78**: 2483–2487

Dion AS, Girardi AJ, Williams CC and Pomenti AA (1986) Serologic responses to murine mammary tumor virus (MuMTV) in MuMTV-exposed laboratory personnel. *J Natl Cancer Inst* **76**: 611–619

Dmochowski L, Seman G and Gallager HS (1969) Viruses as possible etiologic factors in human breast cancer. *Cancer* **24**: 1241–1249

Doll R (1969) The geographical distribution of cancer. *Br J Cancer* **23**: 1–8

Doolittle RF, Feng DF, Johnson MS and McClure MA (1989) Origins and evolutionary relationships of retroviruses. *Q Rev Biol* **64**: 1–30

Forsyth A (1985) *Mammals of the Canadian Wild*. Camden House Publishing: London

Fukui K, Noma T, Takeuchi K, Kobayashi N, Hatanaka M and Honjo T (1983) Origin of adult T cell leukemia virus: implication for its zoonosis. *Mol Biol Med* **1**: 447–456

Gecan JS, Thrasher JL, Eisenberg W and Brickey PM (1980) Rodent excreta contamination and insect damage of wheat. *J Food Protection* **43**: 203–204

Golovkina TV, Jaffe AB and Ross SR (1994) Coexpression of exogenous and endogenous mouse mammary tumor virus RNA in vivo results in viral recombination and broadens the virus host range. *J Virol* **68**: 5019–5026

Haga S, Imai S, Okumoto M, Mori N, Nagano K, Nishino T, Yamamoto H, Nishio M, Sasaki IM, Enami J and Sarkar NH (1994) Detection of mouse mammary tumor virus related sequence in spontaneous human mammary tumor tissue. *Int J Oncol* **5**: 368

Holder WD, Jr, Peer GW, Bolognesi DP and Wells SA, Jr (1976) Antibody reacting with mouse mammary tumor virus in serum of breast carcinoma patients. *Surg Forum* **27**: 102–104

Holmes MD, Hunter DJ, Colditz GA, Stampfer MJ, Hankinson SE, Speizer FE, Rosner B and Willett WC (1999) Association of dietary intake of fat and fatty acids with risk of breast cancer. *JAMA* **281**: 914–920

Howard DK and Schlom J (1980) Isolation of a series of novel variants of murine mammary tumor viruses with broadened host ranges. *Int J Cancer* **25**: 647–654

Huguet EL, McMahon JA, McMahon AP, Bicknell R and Harris AL (1994) Differential expression of human *Wnt* genes 2, 3, 4, and 7B in human breast cell lines and normal and disease states of human breast tissue. *Cancer Res* **54**: 2615–2621

 © 2000 Cancer Research Campaign

Hunter DJ, Spiegelman D, Adami HO, Beeson L, van den Brandt PA, Folsom AR, Fraser GE, Goldbohm RA, Graham S, Howe GR and et al. (1996) Cohort studies of fat intake and the risk of breast cancer – a pooled analysis. *N Engl J Med* **334**: 356–361

Hunter DJ, Hankinson SE, Laden F, Colditz GA, Manson JE, Willett WC, Speizer FE and Wolff MS (1997) Plasma organochlorine levels and the risk of breast cancer. *N Engl J Med* **337**: 1253–1258

Imai S (1996) Mouse mammary tumor virus and mammary tumorigenesis in wild mice. *Pathol Int* **46**: 919–932

Imai S, Okumoto M, Iwai M, Haga S, Mori N, Miyashita N, Moriwaki K, Hilgers J and Sarkar NH (1994) Distribution of mouse mammary tumor virus in Asian wild mice. *J Virol* **68**: 3437–3442

Iscovich J and Howe GR (1998) Cancer incidence patterns (1972–91) among migrants from the Soviet Union to Israel. *Cancer Causes Control* **9**: 29–36

Jouvin-Marche E, Cazenave PA, Voegtle D and Marche PN (1992) V β 17 T-cell deletion by endogenous mammary tumor virus in wild-type-derived mouse strain. *Proc Natl Acad Sci USA* **89**: 3232–3235

Kozak C, Peters G, Pauley R, Morris V, Michalides R, Dudley J, Green M, Davisson M, Prakash O, Vaidya A and et al. (1987) A standardized nomenclature for endogenous mouse mammary tumor viruses. *J Virol* **61**: 1651–1654

Labrecque N, McGrath H, Subramanyam M, Huber BT and Sekaly RP (1993) Human T cells respond to mouse mammary tumor virus-encoded superantigen: V β restriction and conserved evolutionary features. *J Exp Med* **177**: 1735–1743

Lasfargues EY, Lasfargues JC, Dion AS, Greene AE and Moore DH (1976*a*) Experimental infection of a cat kidney cell line with the mouse mammary tumor virus. *Cancer Res* **36**: 67–72

Lasfargues EY, Vaidya AB, Lasfargues JC and Moore DH (1976*b*) In vitro susceptibility of mink lung cells to the mouse mammary tumor virus. *J Natl Cancer Inst* **57**: 447–449

Lasfargues EY, Coutinho WG and Dion AS (1979) A human breast tumor cell line (BT-474) that supports mouse mammary tumor virus replication. *In Vitro* **15**: 723–729

Liu B, Wang Y, Holland JF and Pogo BGT (1999) Sequences homologous to mouse mammary tumor virus in human breast cancer. *Proc Am Assoc Cancer Res* **40**: 444 (Abstract 2934)

Lopez DM, Parks WP, Silverman MA and Distasio JA (1981) Lymphoproliferative responses to mouse mammary tumor virus in lymphocyte subsets of breast cancer patients. *J Natl Cancer Inst* **67**: 353–358

Lushnikova AA, Kriukova IN, Malivanova TF, Makhov PB and Polevaia EB (1998) Correlation between expression of antigen immunologically related to gp52 MMTV and transcription of homologous ENV MMTV DNA sequences in peripheral blood lymphocytes from breast cancer patients [in Russian]. *Mol Gen Mikrobiol Virusol* **3**: 33–36

Miller AB and Gaudette LA (1996) Breast cancer in Circumpolar Inuit 1969–1988. *Acta Oncol* **35**: 577–580

Mills JN and Childs JE (1998) Ecologic studies of rodent reservoirs: their relevance for human health. *Emerg Infect Dis* **4**: 529–537

Nishio M, Xu L, Sasaki M, Haga S, Okumoto M, Mori N, Sarkar NH, Acha-Orbea H, Enami J and Imai S (1994) Complete nucleotide sequence of mouse mammary tumor virus from JYG Chinese wild mice: absence of bacterial insertion sequences in the cloned viral *gag* gene. *Breast Cancer* **1**: 89–94

Nusse R (1991) Insertional mutagenesis in mouse mammary tumorigenesis. *Curr Top Microbiol Immunol* **171**: 43–65

Parkin DM, Whelan SL, Raymond L and Young J (1997) *Cancer Incidence in Five Continents*. Vol. **VII**. IARC Scientific Publications: Lyon.

Pogossiantz H (1956) Some data on the experimental studies of the nature of mammary cancer in mice carried out in the Soviet Union. *Acta Unio internationalis contra Cancrum* **12**: 690–700

Poon MC, Tomana M and Niedermeier W (1983) Serum antibodies against mouse mammary tumor-virus-associated antigen detected nine months before appearance of a breast carcinoma. *Ann Intern Med* **98**: 937–938

Prager EM, Sage RD, Gyllensten U, Thomas WK, Hübner R, Jones CS, Noble L, Searle JB and Wilson AC (1993) Mitochondrial DNA sequence diversity and the colonization of Scandinavia by house mice from East Holstein. *Biol J Linn Soc* **50**: 85–122

Ringold GM, Cardiff RD, Varmus HE and Yamamoto KR (1977) Infection of cultured rat hepatoma cells by mouse mammary tumor virus. *Cell* **10**: 11–18

Rongey RW, Hlavackova A, Lara S, Estes J and Gardner MB (1973) Types B and C RNA virus in breast tissue and milk of wild mice. *J Natl Cancer Inst* **50**: 1581–1589

Sage RD, Atchley WR and Capanna E (1993) House mice as models in systematic biology. *Syst Biol* **42**: 523–561

Stewart T, Tsai SC, Grayson H, Henderson R and Opelz G (1995) Incidence of de-novo breast cancer in women chronically immunosuppressed after organ transplantation. *Lancet* **346**: 796–798

Stewart THM and Heppner GH (1997) Immunological enhancement of breast cancer. *Parasitology* **115**: S141–153

Szabo CI and King MC (1997) Population genetics of BRCA1 and BRCA2. *Am J Hum Genet* **60**: 1013–1020

Tajima K and Cartier L (1995) Epidemiological features of HTLV-I and adult T cell leukemia. *Intervirology* **38**: 238–246

Titus-Ernstoff L, Egan KM, Newcomb PA, Baron JA, Stampfer M, Greenberg ER, Cole BF, Ding J, Willett W and Trichopoulos D (1998) Exposure to breast milk in infancy and adult breast cancer risk. *J Natl Cancer Inst* **90**: 921–924

Traina-Dorge V and Cohen JC (1983) Molecular genetics of mouse mammary tumor virus. *Curr Top Microbiol Immunol* **106**: 35–56

Wang Y, Holland JF, Bleiweiss IJ, Melana S, Liu X, Pelisson I, Cantarella A, Stellrecht K, Mani S and Pogo BG (1995) Detection of mammary tumor virus *env* gene-like sequences in human breast cancer. *Cancer Res* **55**: 5173–5179

Wei WZ, Gill RF and Wang H (1993) Mouse mammary tumor virus associated antigens and super antigens – immuno-molecular correlates of neoplastic progression. *Semin Cancer Biol* **4**: 205–213

Winter H, Cheng KK, Cummins C, Maric R, Silcocks P and Varghese C (1999) Cancer incidence in the south Asian population of England (1990–1992). *Br J Cancer* **79**: 645–654

Wiseman CL, Bowen JM, Davis JW, Hersh EM, Brown BW and Blumenschein GR (1980) Human lymphocyte blastogenesis responses to mouse mammary tumor virus. *J Natl Cancer Inst* **64**: 425–430

Ziegler RG, Hoover RN, Pike MC, Hildesheim A, Nomura AM, West DW, Wu-Williams AH, Kolonel LN, Horn-Ross PL, Rosenthal JF et al (1993) Migration patterns and breast cancer risk in Asian-American women. *J Natl Cancer Inst* **85**: 1819–1827

Zotter S (1983) Widespread occurrence in mammals of antibodies reactive to intracytoplasmic A particles of the mouse mammary tumor virus. *Arch Geschwulstforsch* **53**: 315–319

*© 2000 Cancer Research Campaign*
